# Übersetzung und sprachliche Validierung des modifizierten-Short QUestionnaire to Assess Health-enhancing physical activity (mSQUASH) ins Deutsche für Patienten mit axialer Spondyloarthritis (axSpA)

**DOI:** 10.1007/s00393-024-01508-9

**Published:** 2024-05-08

**Authors:** David Kiefer, Kristina Vaupel, Uta Kiltz, Ludwig Hammel, Yvonne M. van der Kraan, Suzanne Arends, Xenofon Baraliakos

**Affiliations:** 1 Ruhr-Universität Bochum, Herne, Deutschland; 2 Rheumazentrum Ruhrgebiet, Claudiusstr. 45, 44649 Herne, Deutschland; 3 Clinical Science Scholarship, Harvard Medical School, Boston, MA USA; 4 Deutsche Vereinigung Morbus Bechterew e. V., Schweinfurt, Deutschland; 5 Department of Rheumatology and Clinical Immunology, University of Groningen, University Medical Center Groningen, Groningen, Niederlande

**Keywords:** Bewegung, Körperliche Aktivität, Sport, Fragebogen, Patientenbezogenes Ergebnis, Mobility, Physical activity, Sport, Questionnaire, Patient-reported outcome

## Abstract

**Hintergrund:**

Patienten mit axialer Spondyloarthritis (axSpA) leiden oft unter chronischen Schmerzen und entzündlichen Veränderungen, die zu Einschränkungen der körperlichen Funktionsfähigkeit, eingeschränkter Beweglichkeit und verminderter körperlicher Aktivität führen können. Der „Modified-Short QUestionnaire to Assess Health-enhancing physical activity (mSQUASH)“, ein Fragebogen zur Erfassung gesundheitsfördernder körperlicher Aktivität, wurde entwickelt, um die tägliche körperliche Aktivität bei Patienten mit axSpA zu messen.

**Ziel:**

Es erfolgten eine Übersetzung, interkulturelle Anpassung und linguistische Validierung des originalen mSQUASH ins Deutsche für Patienten mit axSpA.

**Methoden:**

Der ursprüngliche mSQUASH-Fragebogen wurde in einem mehrstufigen Prozess (Beaton-Methode) der interkulturellen Anpassung mit Vorwärts-Rückwärts-Übersetzungen vom Niederländischen ins Deutsche durch zweisprachige niederländisch-deutsche Laien und Experten übersetzt. Verbleibende Abweichungen wurden in einer Expertengruppe besprochen, was zu einer vorläufigen deutschen Version führte. Kognitive Befragungen von Patienten mit axSpA mit unterschiedlichen soziodemografischen Hintergründen führten dann zur finalen deutschen Version.

**Ergebnisse:**

Während der Übersetzungen wurden geringfügige Abweichungen hauptsächlich in Bezug auf Formalitäten, semantische Fehler und Syntax festgestellt. Diese wurden angepasst, was zu geringfügigen Änderungen in der Formulierung führte. Die vorläufige deutsche Version wurde durch kognitive Befragungen mit 10 Patienten mit axSpA validiert, wodurch ihre linguistische Validität und Äquivalenz zur niederländischen Version bestätigt wurde.

**Diskussion:**

Insgesamt bestätigte die Untersuchung, dass der finale deutsche mSQUASH ein geeignetes Instrument zur Erfassung der täglichen körperlichen Aktivität ist. Daher kann der vorliegende Fragebogen als Messinstrument in klinischen Studien und in der klinischen Praxis bei deutschsprachigen Patienten mit axSpA eingesetzt werden. Dies kann länderübergreifende Vergleiche ermöglichen und erweitert den Nutzen des Fragebogens über Sprachbarrieren hinweg.

**Zusatzmaterial online:**

Die Online-Version dieses Beitrags (10.1007/s00393-024-01508-9) enthält die deutsche Version des mSQUASH.

## Abstract (en)

**Background:**

Patients with axial spondyloarthritis (axSpA) often experience chronic pain and inflammation, resulting in physical impairments, reduced mobility and decreased physical activity. The modified short questionnaire to assess health-enhancing physical activity (mSQUASH) was developed to assess daily physical activity in patients with axSpA.

**Objective:**

To translate, cross-culturally adapt and linguistically validate the original mSQUASH into German for patients with axSpA.

**Methods:**

The original mSQUASH was translated from Dutch into German using a multistep process (Beaton method) with forward–backward translations into German by bilingual Dutch-German lay people and experts. Any remaining discrepancies were resolved by a scientific committee, resulting in a prefinal German version. Field testing with cognitive debriefing interviews with patients with axSpA from diverse backgrounds led to a final German version.

**Results:**

Minor discrepancies, primarily related to formalities, semantic errors and syntax were found during translations. These were addressed, resulting in slight wording modifications. The prefinal German version was validated through cognitive debriefing by 10 patients with axSpA, confirming its linguistic validity and equivalence to the Dutch version.

**Conclusion:**

Overall, this study confirmed the final German mSQUASH as a comprehensive measurement instrument for daily physical activity. It can now be used as a patient-reported outcome by German patients with axSpA. This can enable cross-linguistic comparisons and expanding its utility across language barriers.

Die axiale Spondyloarthritis (axSpA) ist eine chronische rheumatische Erkrankung, die durch chronische Rückenschmerzen sowie axiale, periphere und extramuskuloskeletale Manifestationen gekennzeichnet ist [29, 30]. Charakteristische Symptome wie entzündliche Rückenschmerzen treten oft bereits im frühen Erwachsenenalter auf. Im Verlauf der Erkrankung sind viele Patienten in ihren beruflichen und freizeitlichen Aktivitäten eingeschränkt [6, 19]. Die Erkrankung ist durch Entzündungen und strukturelle Schäden wie Knochenneubildung im Achsenskelett gekennzeichnet, die bis hin zur vollständigen Ankylose führen kann. Beide, entzündliche Veränderungen und strukturelle Schäden, verursachen Einschränkungen der körperlichen Funktionsfähigkeit und der Wirbelsäulenbeweglichkeit [11, 19, 21, 36]. Neben medikamentösen Therapien ist regelmäßige körperliche Aktivität ein Eckpfeiler in der Behandlung von Patienten mit axSpA. In den aktuellen European League Against Rheumatism(EULAR)-Empfehlungen für körperliche Aktivität werden die Bedeutung und die Umsetzung hervorgehoben [27]. Zudem werden in internationalen und nationalen Empfehlungen für die Behandlung von Patienten mit axSpA Physiotherapie und körperliche Aktivität als Behandlungsmodalitäten betont, die unmittelbar nach der Diagnosestellung begonnen und während des gesamten Krankheitsverlaufs fortgesetzt werden sollten [12, 14, 25].

Basierend auf den internationalen Empfehlungen der Weltgesundheitsorganisation (WHO) für die Allgemeinbevölkerung veröffentlichte die EULAR Empfehlungen zur körperlichen Aktivität für Patienten mit entzündlichen Erkrankungen und degenerativen Erkrankungen [27]. Diese Empfehlungen bieten evidenzbasierte Anleitungen zur Verschreibung, Durchführung und Umsetzung von körperlicher Aktivität und Übungen für Patienten mit entzündlichen und degenerativen Erkrankungen einschließlich axSpA [15, 22, 24].

Eine kürzlich durchgeführte Metaanalyse unterstrich, dass unabhängig von der Art der Übungen positive Auswirkungen auf die Krankheitsaktivität, die Funktionsfähigkeit und die Mobilität bestehen [26]. Es ist bekannt, dass sich Physiotherapie und intensive körperliche Aktivität direkt positiv auf die Krankheitsaktivität wie auch die Beweglichkeit auswirken kann [17, 31, 32].

In dem aktuellen von der ASAS empfohlenen Core-Set von Messinstrumenten für klinische Studien wurden Messungen zur Wirbelsäulenbeweglichkeit ebenfalls mit aufgenommen. Ein Instrument zur Messung der körperlichen Aktivität wurde jedoch nicht benannt [23].

Zur Messung der körperlichen Aktivität, der körperlichen Funktionsfähigkeit sowie der Beweglichkeit stehen verschiedene Ansätze zur Verfügung. Es gibt zum einen Patientenfragebögen wie den mSQUASH und den „International Physical Activity Questionnaire (IPAQ)“ [4, 8, 39] zur Messung der körperlichen Aktivität; der Bath Ankylosing Spondylitis (AS) Functional Index (BASFI) steht zur Messung der körperlichen Funktionsfähigkeit zur Verfügung. Zum anderen gibt es metrische Messmethoden der Wirbelsäulenbeweglichkeit mittels des Bath AS Metrology Index (BASMI) [10]. Darüber hinaus stehen Leistungstests wie der AS Performance Index (ASPI) [35] sowie elektronische Hilfsmittel wie Akzelerometer oder Pedometer zur Verfügung [18, 33].

Der SQUASH-Fragebogen wurde vom niederländischen Nationalen Institut für Volksgesundheit und Umweltstatistik zur Erfassung der körperlichen Aktivität bei Erwachsenen entwickelt und in der allgemeinen niederländischen Bevölkerung validiert [39]. Anhand des Outcome Measures in Rheumatology(OMERACT)-Filters wurden die Inhalts- und Konstruktvalidität sowie die Test-Retest-Reliabilität des SQUASH analysiert.

Im Vergleich zum IPAQ zeigte der SQUASH eine gute Test-Retest-Reliabilität und scheint zudem besser geeignet zu sein, um die tägliche körperliche Aktivität von Patienten mit axSpA zu erfassen, da der SQUASH diese unkompliziert und detaillierter misst [3, 7]. Um die Erfassung der täglichen körperlichen Aktivität speziell bei Patienten mit axSpA zu verbessern, wurde der ursprüngliche SQUASH-Fragebogen von einem Expertenteam, bestehend aus Patienten mit axSpA und Gesundheitsfachleuten in den modifizierten SQUASH (mSQUASH) umgewandelt [7]. Diese Anpassungen resultierten in einem leicht anwendbaren, validen und zuverlässigen Fragebogen zur Beurteilung der täglichen körperlichen Aktivität bei Patienten mit axialer Spondyloarthritis (axSpA), der sowohl in der Forschung als auch in der täglichen klinischen Praxis verwendet werden kann [7].

Der mSQUASH-Fragebogen ist derzeit jedoch nicht auf Deutsch verfügbar. Daher war das Ziel dieser Studie, den niederländischen mSQUASH-Fragebogen ins Deutsche zu übersetzen und interkulturell anzupassen sowie einen Feldtest mit deutschen Patienten mit axSpA durchzuführen.

## Methoden

### Der SQUASH-Fragebogen

Der mSQUASH-Fragebogen bezieht sich auf tägliche körperliche Aktivität in einer durchschnittlichen Woche des letzten Monats und umfasst Aktivitäten in 4 Bereichen: Pendleraktivitäten (von/zur Arbeit, Schule oder zum Studium, Einkaufen), Aktivitäten bei der Arbeit und in der Schule, Hausarbeiten und Freizeitaktivitäten/Sport [7]. Für jede Aktivität wird die Anzahl der Tage pro Woche, die durchschnittliche Zeit und die Intensität (langsam/mäßig/schnell oder leicht/mäßig/schwer) erfragt. Zur Berechnung der Aktivitätspunkte werden die Gesamtzeit pro Aktivität, die wahrgenommene Intensität und die MET(„metabolic equivalent of task“)-Werte des Ainsworth-Kompendiums berücksichtigt [1]. Dabei steht MET für die metabolische Äquivalenz einer Tätigkeit und ist definiert als 1 kcal/kg/h sowie als Sauerstoffaufnahme in ml/kg/min und entspricht daher in etwa den Energie- und Sauerstoffkosten für ruhiges Sitzen [1]. Der Gesamtscore des mSQUASH-Fragebogens ergibt sich aus der Summe aller Aktivitätspunkte innerhalb der spezifischen Bereiche [7].

### Übersetzung und kulturübergreifende Anpassung des mSQUASH

Zur Übersetzung und kulturübergreifenden Anpassung des mSQUASH in die deutsche Sprache wurden die aktuellen internationalen Empfehlungen mit Vorwärts-Rückwärts-Übersetzungen gemäß der Beaton-Methode verwendet [5].

#### Auswahl der Teilnehmer.

Für die Übersetzung wurden 3 Gruppen für die Vorwärts-Rückwärts-Übersetzungen sowie für die kognitiven Befragungen zusammengestellt.

#### Laienbeteiligte.

Zwei Patienten mit axSpA wurden mit Unterstützung der deutschen Patientenorganisation „Deutsche Vereinigung Morbus Bechterew e. V.“ rekrutiert, ohne dass ihnen kontextbezogene Informationen zum Fragebogen zur Verfügung gestellt wurden. Beide Patienten übersetzten die Fragebögen unabhängig voneinander. Die Patienten waren zweisprachig oder sogar muttersprachlich in Deutsch und Niederländisch und ≥ 18 Jahre.

#### Expertengruppe.

Ein Rheumatologe und ein nichtärztlicher Wissenschaftler.

#### Patienten zur kognitiven Befragung.

Es wurde eine hinsichtlich Alter, Geschlecht und soziodemografischen Daten und Bildungsniveau heterogene Gruppe von 10 erwachsenen Patienten mit axSpA rekrutiert. Die Einschlusskriterien waren zum einem die fachärztliche Diagnose einer axSpA und zum anderen, fließend deutsch zu sprechen. Neben demografischen Daten wurden folgende Assessments erhoben: Die Krankheitsaktivität wurde mit dem AS Disease Activity Score (ASDAS) und dem Bath AS Disease Activity Index (BASDAI), die körperliche Funktionsfähigkeit mit dem BASFI erfasst. Zur globalen Funktionsfähigkeit und Gesundheit wurden der Short Form Health Survey 36 (SF36) sowie der ASAS Health Index (ASAS HI) hinzugezogen, um die Teilnehmer zu charakterisieren ([9, 16, 20, 28, 37, 38]; Tab. 1 und 2).

**Tab. 1:** Demografische Daten der einzelnen Patienten aus der kognitiven Befragung

Patient	Alter^a^	Geschlecht^a^	BMI	Beschäftigungsstatus^a^	Erwerbstätigkeitsdauer (Jahre)	Wochenarbeitszeit (h)	Höchster Bildungsgrad^a^
1	43,2	Weiblich	32,5	3	16	54	2
2	53,2	Männlich	26,6	1	36	40	2
3	56,2	Männlich	25,4	3	36	40	2
4	50,0	Männlich	25,9	1	35	37,5	2
5	35,4	Männlich	23,5	1	1	40	4
6	52,8	Weiblich	32,9	2	37	18	1
7	42,3	Weiblich	24,2	1	12	40	4
8	63,0	Männlich	24,6	3	47	40	2
9	55,8	Männlich	27,1	1	38	50	2
10	59,6	Männlich	19,8	3	26	40	2
Mittelwerte (SD)	51,2 (8,5)	30 % Frauen	26,3 (4,0)	–	28,4 (14,3)	37,6 (10,5)	–

Beschäftigungsstatus: 0: Arbeitssuchend, 1: Vollzeit, 2: Teilzeit (inklusive krankheitsbedingte Teilzeit), 3: Verrentet/Frührente, da dauerhaft krank; höchster Bildungsgrad: 0 = Grundschule, 1 = mittlere Reife/Realschulabschluss, 2 = Fachhochschulreife/Berufsausbildung, 3 = Uniabschluss < 4 Jahre, 4 = Uniabschluss > 4 Jahre

*StDev* Standardabweichung, *BMI* Body Mass Index

^a^Ergebnisse als Mittelwerte

**Tab. 2:** Klinische Daten der einzelnen Patienten aus der kognitiven Befragung

Patient	r-/nr axSpA	HLA-B27^a^	Symptomdauer (Jahre)	Schmerzen NRS (0–10)	CRP (mg/dl)	ASDAS-CRP	BASDAI	BASFI	SF36 (%)			ASAS HI	mSQUASH, Aktivitätswert
									MCS	PCS	Total		
1	r‑axSpA	Positiv	7,3	1,0	0,1	3,5	3,03	9,30	29,0	4,0	16,5	12,0	3510
2	r‑axSpA	Negativ	15,3	7,0	0,2	2,5	5,25	5,90	78,3	17,5	47,9	10,0	9156
3	nr-axSpA	Positiv	5,3	7,5	0,2	1,7	3,05	4,20	59,4	15,0	37,2	11,0	304
4	r‑axSpA	Negativ	8,1	7,0	2,2	4,1	3,60	0,80	76,4	65,0	70,7	3,0	8808
5	r‑axSpA	Positiv	6,1	7,0	0,1	2,4	3,80	3,40	77,8	63,8	70,8	6,0	2517
6	r‑axSpA	Positiv	5,1	7,5	0,6	3,5	6,81	5,50	28,8	31,6	30,2	12,0	4194
7	r‑axSpA	Positiv	7,1	6,0	0,1	2,0	4,33	4,40	50,0	75,0	62,5	9,0	8291
8	nr-axSpA	Positiv	34,2	8,0	0,1	3,1	4,73	8,40	66,6	36,9	51,8	10,0	17.454
9	nr-axSpA	Positiv	23,2	7,0	1,4	3,6	5,28	5,40	76,1	23,8	50,0	8,0	966
10	nr-axSpA	Negativ	25,6	7,0	0	3,6	9,65	9,10	24,5	21,9	23,2	13,0	16.892
Mittelwerte (SD)	40 % r‑axSpA	70 % HLA-B27 pos	13,7 (10,4)	6,5 (2,0)	0,5 (0,7)	3,0 (0,8)	4,95 (2,02)	5,64 (2,69)	56,7 (13,7)	35,5 (20,4)	46,1 (17,1)	8,7 (3,1)	7209,2 (6125,4)

*SD* Standardabweichung, *r‑axSpA* röntgenologische axSpA, *nr-axSpA* nichtröntgenologische axSpA, *HLA-B27* humanes Leukozytenantigen B27, *NRS* „numerical rating scale“ (0 = keine Schmerzen, 10 = schlimmste Schmerzen), *CRP* C-reaktives Protein, *ASDAS* Ankylosing Spondylitis Disease Activity Score, *BASDAI* Bath Ankylosing Spondylitis Disease Activity Index, *BASFI* Bath Ankylosing Spondylitis Functional Index, *SF36* Short Form 36, *MCS* Mental Component Score, *PCS* Physical Component Score, *ASAS HI* Assessment for Ankylosing Spondylitis Society Health Index, *mSQUASH* modifizierter-Short QUestionnaire to Assess Health-enhancing physical activity

^a^Ergebnisse als Mittelwerte

#### Kulturübergreifende Anpassung: Übersetzungen und Expertenbewertung.

Nach der von Beaton vorgeschlagenen Methodik verlief der gesamte Prozess der kulturübergreifenden Anpassung als ein Feedback-Loop aus Vorwärtsübersetzung (Schritt 1) und Rückwärtsübersetzung (Schritt 3). Dies umfasste Schritte wie die Identifizierung, Diskussion und Klärung von sprachlichen Bedeutungsunterschieden, die Synthese und die Bildung von Konsens (Schritte 2 und 4). Schritt 4 wurde durch die Überprüfung eines Expertengremiums abgeschlossen. Die final konsentierte deutsche Version wurde anschließend von einer Patientengruppe mittels kognitiver Befragungen in Feldtests überprüft (Schritt 5).

#### Vorwärtsübersetzung des originalen niederländischen mSQUASH-Fragebogens ins Deutsche (Schritte 1 und 2).

Nach der Rekrutierung der Laienbeteiligten erhielt jeder Teilnehmer die Originalversion des mSQUASH-Fragebogens auf Niederländisch (Schritt 1, Vorwärtsübersetzung). Sie wurden gebeten, ihre eigene Übersetzung in die deutsche Sprache vorzulegen. Diese Übersetzungen umfassten den gesamten Fragebogen einschließlich der Einführungstexte, Anleitungstexte, Beispiele und Antworten. Die von den Laienbeteiligten vorgelegten Vorwärtsübersetzungen wurden von der Expertengruppe auf Unterschiede zwischen den Übersetzungen überprüft (Schritt 2). Dissonanzen wurden mit den Laienbeteiligten diskutiert, und Konsens wurde gefunden. Eine gemeinsame, harmonisierte und synthetisierte Übersetzung wurde für die anschließende Rückwärtsübersetzung verwendet.

#### Rückwärtsübersetzung und Überprüfung durch das Expertengremium (Schritte 3 und 4).

Die Rückwärtsübersetzungen wurden abschließend von der Expertengruppe bewertet, um die Konsistenz in beiden Übersetzungsprozessen sicherzustellen und zumindest die semantische, idiomatische, erfahrungsbezogene und konzeptuelle Äquivalenz zwischen der Ausgangs- und der übersetzten Zielfragebogenversion aufrechtzuerhalten. Die Expertengruppe finalisierte eine konsentierte Vorfinalversion des deutschen mSQUASH, die zur linguistischen Validierung in Einzelinterviews genutzt wurde. Ergebnisse dieser Einzelinterviews wurden von der Expertengruppe in die Vorfinalversion eingearbeitet und sie somit finalisiert.

#### Linguistische Validierung: Feldtests mit teilstrukturierten Einzelinterviews zur kognitiven Befragung (Schritt 5).

Um die linguistische Validierung der Vorfinalversion des deutschen mSQUASH sicherzustellen, wurde eine kognitive Befragung mit einer Gruppe von 10 erwachsenen Patienten mit axSpA (≥ 18 Jahre) durchgeführt.

Dies diente dazu, während der Beantwortung von Fragebögen die Qualität und Verlässlichkeit der Daten zu gewährleisten.

Die Patienten wurden gebeten, den deutschen mSQUASH auszufüllen. Zusätzlich wurde eine kognitive Befragung mit einem nichtärztlichen Wissenschaftler durchgeführt. Jeder Teilnehmer füllte den mSQUASH aus und wurde nach der Vollständigkeit, Verständlichkeit und Akzeptanz aller Fragen und Antworten befragt. Die kognitive Befragung sollte Informationen über das Verständnis der konsentierten Übersetzungsversion erfassen sowie sicherstellen, dass die Befragten den Fragebogen genauso verstehen wie das Original. Zudem sollte überprüft werden, dass die Einhaltung aller 4 Äquivalenzebenen zur niederländischen Originalversion gewährleistet ist (semantische, idiomatische, erfahrungsbezogene und konzeptuelle Äquivalenz nach Beaton). Letztlich sollten so potenziell verwirrende Ausdrücke oder Elemente erkannt werden.

Für diese Studie liegt eine Genehmigung der Ethikkommission der Ruhr-Universität Bochum (Registernummer 21-7179) vor.

## Ergebnisse

### Vorwärtsübersetzung des originalen niederländischen mSQUASH-Fragebogens ins Deutsche.

Die Hauptunterschiede zwischen den beiden Übersetzungen betrafen die Verwendung formeller Sprache im Vergleich zur informellen, leicht verständlichen Sprache.

### Rückübersetzung der vorläufigen deutschen mSQUASH-Version ins Niederländische und anschließende Entwicklung der konsensuellen deutschen Version nach Expertenprüfung.

Bei dem Vergleich der rückübersetzten niederländischen Version mit der niederländischen Originalversion wurden nur geringfügige Unterschiede festgestellt, da im Zwischenschritt der Vorwärtsübersetzung ins Deutsche bereits eine gute Übereinstimmung in der Konsensversion erzielt wurde.

### Kognitive Befragung der vorläufigen deutschen Konsensusversion des mSQUASH durch Patienten mit axSpA in Einzelinterviews

Zehn Patienten mit axSpA wurden eingeschlossen. Die Teilnehmer zeigten sich heterogen in Bezug auf demografische Merkmale wie Alter, Geschlecht, Familienstand, Krankheitsdauer, Bildungsgrad, krankheitsspezifische Assessments und andere soziodemografische Daten (Tab. 1 und 2).

Die Patienten wurden im Rahmen der kognitiven Überprüfung in Einzelinterviews nach Relevanz und der Verständlichkeit des vorläufigen Fragebogens gefragt. Insgesamt benötigten die Patienten durchschnittlich 5,0 min (± 1,8) zum Ausfüllen des Fragebogens.

Um die Lesart des Fragebogens zu verbessern, wurden einige semantische Änderungen durchgeführt, ohne jedoch die Struktur oder den inhaltlichen Kontext des Fragebogens zu verändern. Insgesamt gaben die Teilnehmer an, dass die Anweisungen des Fragebogens klar und leicht verständlich seien und hielten die Fragen für klar formuliert. Zudem wurden keine Wörter oder ganze Fragen als kulturell unangemessen angesehen. Die Wortwahl war eindeutig, und die Fragen waren mehrheitlich leicht zu beantworten.

Einige der semantischen Änderungen werden im Folgenden beschrieben: So wurde „anderer Transport“ in „andere Ziele“ geändert; „Körperliche Aktivität bei der Arbeit oder in der Schule“ wurde in „körperliche Aktivität bei der Arbeit (bezahlt/unbezahlt) oder Schule/Studium“ geändert. Im Feld „Sport und Bewegung“ wurde „Tanzen“ als Beispiel hinzugefügt. In der kognitiven Befragung gaben 70 % Teilnehmer an, dass sie unter „Wandern“ einen mehrstündigen, mindestens mäßig anstrengenden Weg in der Natur verstehen, daher erwies sich die Übersetzung semantisch als nicht haltbar. Da auch ein Spaziergang gemeint sein kann, wurde in der finalen deutschen Version das niederländische „wandelen“ in „spazieren gehen“ übersetzt. Zudem wurde in der Anleitung und im Folgenden der Begriff „Tätigkeiten“ durch „Aktivitäten“ ersetzt, da dies für die 80 % der Teilnehmer der treffendere Begriff war. Insgesamt wurden alle Fragen von den Patienten für relevant eingestuft, um die tägliche körperliche Aktivität zu bewerten. Zudem gaben alle Patienten an, dass keine Aspekte der körperlichen Aktivität im Fragenbogen fehlten.

Es wurde zudem angegeben, dass einige Fragen zur individuellen Lebenssituation nicht passen würden. So erklärte die Mehrheit der Teilnehmer, nicht mit dem Fahrrad zu Arbeit zu fahren oder dorthin zu Fuß zu gehen. Daraufhin wurde die Antwortmöglichkeit „nicht zutreffend“ in den Fragebogen aufgenommen.

Einige weitere Kommentare zum Fragebogen, inhaltlich unabhängig von der kulturellen und sprachlichen Validierung, wurden von den Teilnehmern abgegeben: So erklärten 70 % der Teilnehmer, dass der Fragebogen sie darauf aufmerksam machte, welche Sportarten sie aufgrund ihrer Erkrankung nun nicht mehr oder nur sehr eingeschränkt ausführen können; des Weiteren wurde angeführt, dass ihnen grundsätzlich Hilfestellungen für den eigenen Umgang mit körperlicher Aktivität fehlen würden.

Zudem wurde angegeben, dass das Fragebogenergebnis keine Assoziierung mit ihrer Krankheitsaktivität ermögliche oder keine Aussage zum erforderlichen Maß an sportlicher Aktivität beispielsweise zur Schubvermeidung träfe. Da der Fragebogen nur die Aktivitäten einer normalen Woche im letzten Monat erfragt, wurde ebenfalls angemerkt, dass viele Aspekte des mSQUASH-Fragebogens nicht auf ihre Situation zutreffen würden oder dass die Aktivitäten von ihnen nur saisonal bzw. je nach Wetterlage ausgeführt werden würden. Der Fragebogen würde diese Differenzierung aber nicht ermöglichen.

Die endgültige deutsche Version des mSQUASH findet sich im Online-Zusatzmaterial.

## Diskussion

Der mSQUASH-Fragebogen konnte mittels Vorwärts- und Rückwärtsübersetzung erfolgreich ins Deutsche übersetzt werden [5]. Die Untersuchung identifizierte geringfügige Abweichungen während des Übersetzungsprozesses, hauptsächlich in Bezug auf Formalitäten, semantische Fehler und Syntax. Diese wurden jedoch im Laufe der Diskussion und Konsensfindung zwischen den Laienbeteiligten und der Expertengruppe erfolgreich angepasst. Der Fokus lag darauf, eine konsentierte Version zu erstellen, die die semantische, idiomatische, erfahrungsbezogene und konzeptuelle Äquivalenz zur Originalversion in Niederländisch aufrechterhielt. Während der kognitiven Befragung äußerten einige Teilnehmer dennoch Kritikpunkte bezüglich der Relevanz des Fragebogens für die Beeinflussung ihres individuellen Krankheitsverlaufs. Wie bereits beschrieben [13], zeigte sich auch in dieser Untersuchung, dass ein Teil der Patienten nicht ausreichend über die positiven Effekte von körperlicher Aktivität auf ihre Erkrankung informiert ist. Daher konnten einige Teilnehmer der kognitiven Befragung teilweise keine direkte Verbindung zwischen dem Fragebogen und ihrer Erkrankung herstellen [13]. Die Teilnehmer wünschten sich daher, unabhängig von der Beantwortung des Fragebogens, Hinweise zur Verbesserung ihrer körperlichen Aktivität und der Implementierung aktivitätsfördernder Maßnahmen in ihr eigenes Krankheitsmanagement. Aufklärung und Schulung von Patienten mit axSpA über die Vorteile körperlicher Aktivität sind daher ein wichtiger Aspekt für die Behandlung der Patienten, jedoch nicht Gegenstand und Aufgabe des mSQUASH. Da der mSQUASH ein Messinstrument ist, das Einblick in die Art und Dauer der durchgeführten Aktivitäten gibt, können die Ergebnisse des Fragebogens während einer Beratung mit dem Arzt oder der Ärztin und der Physiotherapeutin oder dem Physiotherapeuten genutzt werden, um Möglichkeiten zur Förderung und Verbesserung der körperlichen Aktivität zu besprechen.

Die Patienten betrieben zum Teil saisonabhängige Sportarten, die sie nicht unter den Beispielen fanden, oder konnten diese aber zum Befragungszeitpunkt aufgrund der Wetterlage nicht ausüben, sodass Patienten mit beiden Begründungen bei dieser Frage „nicht zutreffend“ angaben. Um nicht zutreffende Begründungen – und damit ein „response-bias“ – möglichst zu vermeiden und um die Relevanz des Fragebogens für den Patienten saisonunabhängig zu erhöhen, könnten saisonale Unterschiede durch den mehrmaligen Einsatz des Fragebogens zu verschiedenen Zeitpunkten im Jahr erfasst werden.

Die interkulturelle und sprachliche Validität des finalen deutschen mSQUASH-Fragebogens wurde durch die Methode nach Beaton gewährleistet [5]. Die identifizierten Anpassungen während des Prozesses unterstreichen die Bedeutung der sorgfältigen kulturellen Anpassung von Instrumenten, um ihre Validität und Anwendbarkeit in verschiedenen Sprachkontexten sicherzustellen. Die Ergebnisse dieser Studie legen nahe, dass der deutsche mSQUASH als Instrument zur standarisierten Erfassung der wöchentlichen körperlichen Aktivität bei Patienten mit axSpA verwendet werden kann. Die Übersetzung des mSQUASH ins Deutsche ermöglicht es, den Fragebogen in Deutschland in internationalen klinischen Studien bei Patienten mit axSpA einzusetzen. Eine standardisierte Erfassung und Vergleichbarkeit von Ergebnissen in verschiedenen Sprachregionen wird so ermöglicht. So kann auch die Vergleichbarkeit von Ergebnissen zwischen verschiedenen Patientengruppen und über verschiedene Studien hinweg gewährleistet werden. Es ist bekannt, dass regelmäßige körperliche Aktivität einen entscheidenden Beitrag zur Behandlung und Bewältigung der axSpA-Erkrankung leisten kann [26, 31]. Um körperliche Aktivität auch bei deutschsprachigen Patienten mit axSpA regelmäßig und standardisiert erfassen zu können, ist eine umfassende Validierung des mSQUASH als klinisches Messinstrument erforderlich. Der mSQUASH erfasst sämtliche täglichen Aktivitätsbereiche in unterschiedlichen Intensitäten und ermöglicht so einen direkten Überblick über den Verlauf der täglichen körperlichen Aktivität [3, 7, 34]. Im Hinblick auf Veränderungen in der Häufigkeit der Ausführung oder der Intensität körperlicher Aktivität zeigt der mSQUASH eine gute Sensitivität und Reaktionsfähigkeit, sodass Steigerungen oder Abnahmen vom mSQUASH angezeigt wurden, anschließend durch BASDAI-Veränderungen bestätigt wurden [7]. Wichtig ist jedoch hervorzuheben, dass die Selbstwahrnehmung der Intensität und Dauer der körperlichen Aktivität durch den einzelnen Patienten äußerst subjektiv sein kann. Daher können äußere Faktoren wie Jahreszeiten, Wetter, Urlaub oder Stressniveau, aber auch Faktoren wie Krankheitsaktivität, funktionelle Einschränkungen, Leistungsfähigkeit und Fitnessniveau die Ergebnisse beeinflussen. Dadurch können sich subjektiv ermittelte Werte von objektiv durchgeführten und beispielsweise elektronisch mit einem Akzelerometer oder Pedometer gemessener körperlicher Aktivität unterscheiden [2, 7]. Daher sollten subjektive – und oft überschätzte –, sozial wünschenswerte Intensitätsdaten, die durch den mSQUASH ermittelt werden, sorgfältig interpretiert und durch objektive Messungen der körperlichen Aktivität ergänzt und bestätigt werden [3, 7, 34].

Für das Verständnis und die Implementierung von körperlicher Aktivität in das Krankheits- und Selbstmanagement der Patienten spielen die Förderung und Unterstützung der Patienten durch die behandelnden Ärzte und Physiotherapeuten eine zentrale Rolle [12]. Dies könnte nicht nur langfristig und nachhaltig dazu beitragen, die körperliche Funktionsfähigkeit des Patienten insgesamt zu verbessern, sondern auch die Lebensqualität der Patienten insgesamt zu steigern [17].

Um die interkulturelle Anpassung des mSQUASH ins Deutsche abzuschließen und auch die kritischen Aspekte, die die Teilnehmer der kognitiven Befragung äußerten, besser einzuordnen, sollten im nächsten Schritt die psychometrischen Eigenschaften der deutschen Version sowie qualitative Aspekte wie die Motivation der Patienten beim Ausfüllen des Fragebogens in einer größeren Patientengruppe überprüft werden.

## Fazit für die Praxis

- Der mSQUASH ist ein Fragebogen zur Erfassung der täglichen körperlichen Aktivität von Erwachsenen und ist auch für Patienten mit axSpA validiert.
- Die Übersetzung des mSQUASH ins Deutsche ermöglicht es, den Fragebogen in Deutschland in internationalen klinischen Studien einzusetzen und so eine Vergleichbarkeit von Ergebnissen in verschiedenen Sprachregionen zu gewährleisten.
- Zudem gibt der mSQUASH Einblicke in die Art und Dauer der von Patienten individuell durchgeführten körperlichen Aktivitäten. Daher können die Ergebnisse zur Beratung mit den Ärzten und Physiotherapeuten genutzt werden und so auch Möglichkeiten zur Verbesserung der körperlichen Aktivität individueller Patienten finden.

## Supplementary Information

Finale Version des deutschen mSQUASH
