# Supplementary material for: German translation and linguistic validation of the modified short questionnaire to assess health-enhancing physical activity (mSQUASH) for patients with axial spondyloarthritis (axSpA)
Source: Z Rheumatol. 2024 May 8;84(1):1–9. [Article in German] doi: 10.1007/s00393-024-01508-9 (PMC11779693; doi:10.1007/s00393-024-01508-9)
Supplement: Supplementary file 1 — Finale Version des deutschen mSQUASH [file 393_2024_1508_MOESM1_ESM.pdf]

mSQUASH

Fragebogen – Körperliche Aktivität

Datum:
Name/Studiennummer:
Land:

ANLEITUNG – Bitte lesen Sie die folgenden Hinweise sorgfältig, bevor Sie mit der Beantwortung der Fragen beginnen

Denken Sie an eine normale Woche im letzten Monat. Bitte geben Sie an,

- an wie vielen Tagen pro Woche Sie die untenstehenden Aktivitäten ausführen
- wie viel Zeit Sie jeweils mit den Aktivitäten verbracht haben und
- wie körperlich anstrengend jede dieser Aktivitäten für Sie war.

BEISPIEL

Weg zur Arbeit oder Schule (Hin- und Rückfahrt) (Bezahlte/unbezahlte Arbeit oder Schule/Studium)	Nicht zutreffend	Anzahl Tage pro Woche	Durchschnittliche Dauer pro Tag	Anstrengungsgrad
1. Zu Fuß zur/von der Arbeit oder zur Schule/zum Studium	○	5 Tage	0 Stunden 30 Minuten	☒ Langsam/leicht<br>○ Mäßig<br>○ Schnell/schwer

Erläuterung des Anstrengungsgrades

Langsam/leicht	Ruhiger Herzschlag und ruhige Atmung
Mäßig	Erhöhte Herzschlag und beschleunigte Atmung
Schnell/Schwer	Erhöhte Herzschlag, beschleunigte Atmung und Schwitzen

START FRAGENBOGEN

Weg zur Arbeit oder Schule (Hin- und Rückfahrt) (Bezahlte/unbezahlte Arbeit oder Schule/Studium)	Nicht zutreffend	Anzahl Tage pro Woche	Durchschnittliche Dauer pro Tag	Anstrengungsgrad
1. Zu Fuß zur/von der Arbeit oder zur Schule/zum Studium	○	__ Tage	__Stunden __Minuten	○ Langsam/leicht<br>○ Mäßig<br>○ Schnell/schwer
2. Radfahren zur/von der Arbeit oder zur Schule/zum Studium	○	__ Tage	__Stunden __Minuten	○ Langsam/leicht<br>○ Mäßig<br>○ Schnell/schwer

Andere Ziele (Hin- und Rückfahrt) (Z.B. jemanden besuchen, zum Sportverein oder Supermarkt)	Nicht zutreffend	Anzahl Tage pro Woche	Durchschnittliche Dauer pro Tag	Anstrengungsgrad
3. Zu Fuß zu/von einem anderen Ziel	○	__ Tage	__Stunden __Minuten	○ Langsam/leicht<br>○ Mäßig<br>○ Schnell/schwer
4. Radfahren zu/von einem anderen Ziel	○	__ Tage	__Stunden __Minuten	○ Langsam/leicht<br>○ Mäßig<br>○ Schnell/schwer

Arbeit (bezahlt/unbezahlt) oder Schule/Studium	Nicht zutreffend	Anzahl Stunden pro Woche
5. Wie viele Stunden pro Woche arbeiten Sie oder gehen Sie zur Schule/zum Studium?	○	___Stunden
6. Wie viele von diesen Stunden sind anstrengende Arbeit? (Z.B. regelmäßig schwere Dinge heben)	○	___Stunden

ANLEITUNG

Denken Sie an eine <u>normale Woche im letzten Monat</u>.

Erläuterung des Anstrengungsgrades

Langsam/leicht	Ruhiger Herzschlag und ruhige Atmung
Mäßig	Erhöhte Herzschlag und beschleunigte Atmung
Schnell/Schwer	Erhöhte Herzschlag, beschleunigte Atmung und Schwitzen

**Tätigkeiten im Haushalt**	**Nicht zutreffend**	**Anzahl Tage pro Woche**	**Durchschnittliche Dauer pro Tag**	**Anstrengungsgrad**
7. Leichte und mäßig anstrengende Hausarbeit (Z.B. Kochen, Geschirrspülen, Aufräumen)	○	__ Tage	__Stunden __Minuten	○ Langsam/leicht<br>○ Mäßig<br>○ Schnell/schwer
8. Schwere, anstrengende Hausarbeit (Z.B. Bettwäsche wechseln, kleine Kinder heben, Badezimmer putzen, schwere Lebensmittel heben)	○	__ Tage	__Stunden __Minuten	○ Langsam/leicht<br>○ Mäßig<br>○ Schnell/schwer

**Freizeitaktivitäten**	**Nicht zutreffend**	**Anzahl Tage pro Woche**	**Durchschnittliche Dauer pro Tag**	**Anstrengungsgrad**
9. Spazierengehen	○	__ Tage	__Stunden __Minuten	○ Langsam/leicht<br>○ Mäßig<br>○ Schnell/schwer
10. Fahrradfahren	○	__ Tage	__Stunden __Minuten	○ Langsam/leicht<br>○ Mäßig<br>○ Schnell/schwer
11. Gartenarbeit	○	__ Tage	__Stunden __Minuten	○ Langsam/leicht<br>○ Mäßig<br>○ Schnell/schwer
12. Heimwerken	○	__ Tage	__Stunden __Minuten	○ Langsam/leicht<br>○ Mäßig<br>○ Schnell/schwer
13. Einkaufen	○	__ Tage	__Stunden __Minuten	○ Langsam/leicht<br>○ Mäßig<br>○ Schnell/schwer

**Sport und Bewegung** (Z.B. ins Fitnessstudio gehen, physiotherapeutische Übungen, Laufen, Tennis, Fußball, Schwimmen, Tanzen)	**Nicht zutreffend**	**Anzahl Tage pro Woche**	**Durchschnittliche Dauer pro Tag**	**Anstrengungsgrad**
14. ______________________	○	__ Tage	__Stunden __Minuten	○ Langsam/leicht<br>○ Mäßig<br>○ Schnell/schwer
15. ______________________		__ Tage	__Stunden __Minuten	○ Langsam/leicht<br>○ Mäßig<br>○ Schnell/schwer
16. ______________________		__ Tage	__Stunden __Minuten	○ Langsam/leicht<br>○ Mäßig<br>○ Schnell/schwer
17. ______________________		__ Tage	__Stunden __Minuten	○ Langsam/leicht<br>○ Mäßig<br>○ Schnell/schwer

Ende des Fragebogens.

Würden Sie bitte einmal überprüfen, ob Sie alle Fragen beantwortet haben?

Herzlichen Dank für Ihre Mitarbeit!
